# Establishing the twig method for investigations on pollen characteristics of allergenic tree species

**DOI:** 10.1007/s00484-021-02154-5

**Published:** 2021-05-27

**Authors:** Stephan Jung, Feng Zhao, Annette Menzel

**Affiliations:** 1 TUM School of Life Sciences, Department of Life Science Systems, Technical University of Munich, 85354 Freising, Germany; 2 Helmholtz Zentrum München - Deutsches Forschungszentrum für Gesundheit und Umwelt (GmbH), 85764 Oberschleißheim, Germany; 3 Institute of Advanced Study, Technical University of Munich, 85748 Garching, Germany

**Keywords:** Pollen, Allergy, Climate change, Twig experiment

## Abstract

The twig method in climate chambers has been shown to successfully work as a proxy for outdoor manipulations in various experimental setups. This study was conducted to further establish this method for the investigation of allergenic pollen from tree species (hazel, alder, and birch). Direct comparison under outdoor conditions revealed that the cut twigs compared to donor trees were similar in the timing of flowering and the amount of pollen produced. Cut twigs were able to flower in climate chambers and produced a sufficient amount of pollen for subsequent laboratory analysis. The addition of different plant or tissue fertilizers in the irrigation of the twigs did not have any influence; rather, the regular exchange of water and the usage of fungicide were sufficient for reaching the stage of flowering. In the experimental setup, the twigs were cut in different intervals before the actual flowering and were put under warming conditions in the climate chamber. An impact of warming on the timing of flowering/pollen characteristics could be seen for the investigated species. Therefore, the twig method is well applicable for experimental settings in pollen research simulating, e.g., accelerated warming under climate change.

## Introduction

Under climate change, the relevance of aeroallergens including allergenic pollen is expected to increase (Beggs 2004). Nowadays, pollen-related allergic rhinitis already prevails in up to 40% of the population in Europe (D’Amato et al. 2007). New techniques for the investigation of allergenic pollen are steadily being developed (Sofiev and Bergmann 2013).

The goal of these studies was among others to identify key influences on seasonal patterns and pollen emission, including the effects of climate change (Marselle et al. 2019), and to study pollen characteristics, such as variation in allergic content among different grass species (Jung et al. 2018). It is already known that climate change alters seasonal patterns of pollen, e.g., leading to an earlier start of pollen season (Frei and Gassner 2008; Menzel et al. 2006; Menzel et al. 2020a, b; Rosenzweig et al. 2008). Seasonal patterns and their trends are normally captured by phenological studies and/or longer time series of measured airborne pollen concentrations (e.g., Menzel et al. 2021). In general, pollen characteristics, such as protein content, and allergenicity are obtained from airborne samples using high-volume impactors (Buters et al. 2010; Grewling et al. 2020) or low-volume devices such as multi-vial cyclone samplers (Brennan et al. 2019). However, the acquisition costs for such devices are high and even then, it cannot be ruled out that pollen from more than one individual is collected. Therefore, the study of pollen characteristics of individual species such as birch or hazel usually requires in situ collection of pollen, which should ideally be combined with manipulative experiments on climate change. For the subsequent laboratory analysis, well-established standard methods such as ELISA and Western blot for the determination of allergenic content are used (Bufe et al. 1996; Jung et al. 2018; Schäppi et al. 1999). The necessary in situ collection of pollen is often associated with difficulties, as the pollen has to be stored under suitable conditions immediately after emission. Therefore, pollen collection requires, e.g., air-permeable bags that collect the pollen while protecting it from rain and insects and at the same time likely formation of condensed water has to be prevented. Glassine bags which are commonly used in plant crossing attempts offer air permeability but do not fulfill the other requirements (Hanna 1990; Jung et al. 2018).

Here, the twig method in climate chambers may also meet these requirements for in situ pollen collection allowing at the same time variations in environmental conditions. Thus, this method has the benefit that in the face of climate change different climatic scenarios and their impacts on pollen characteristics can be studied. The twig method has already been well described in previous studies (Basler and Körner 2012; Dantec et al. 2014; Menzel et al. 2020a, b; Primack et al. 2015). Vitasse and Basler (2014) observed for the tree species hornbeam, European beech, and sycamore that the timing of budburst is comparable between cut and uncut twigs from a donor tree. Among others, the twig method can be applied for various phenological observations, e.g., to test the influence of winterly (chilling) temperatures (Laube et al. 2014a) and bud development under elevated air humidity (Laube et al. 2014b).

From the literature, it is known that under warmer spring temperatures, flowering is generally accelerated (Fu et al. 2012; Wang et al. 2020). In the study of Fu et al. (2012), potted saplings from beech, birch, and oak were treated with various temperature manipulations in climate chambers. It revealed that spring warming is more influential on budburst than winter warming and that spring warming (forcing) leads to an earlier budburst. A strong correlation between temperatures in March and the start of pollen season/in situ observed flowering phenology was also observed for birch (Bogawski et al. 2019; Newnham et al. 2013). Another study on birch (Miller-Rushing and Primack 2008) reported that the inflorescence length does not influence the start of flowering. Other studies have shown that the responses to changed climatic conditions are highly species-specific (Laube et al. 2014a; Malyshev 2020; Miller-Rushing and Primack 2008).

The objective of this study was to establish the twig method in climate chambers for the investigation of allergenic pollen. In order to observe the impact of harvesting on flowering and pollen characteristics, an outdoor comparison between cut and uncut inflorescences regarding their pollen production and pollen characteristics was conducted. We examined whether branch experiments in climate chambers can be used as a proxy for outdoor manipulations on pollen. In pollen research, the harvesting of twigs shortly before flowering is required, and then the pollen has to be emitted under controlled conditions in the climate chamber. Furthermore, it was evaluated under controlled conditions in the climate chamber whether the usage of different fertilizers as an additive in the irrigation causes favorable/unfavorable conditions for pollen production and has an impact on pollen characteristics as previous studies suggested for flowering and number of inflorescences (AL-Kahtani and Ahmed 2012; Maksoud 2000).

## Material and methods

### Investigated species and tree selection

We chose the widespread shrub species *Corylus avellana* (hazel) and tree species *Alnus glutinosa* (common alder) and *Betula pendula* (silver birch), which are characterized by a high allergenicity of their pollen and the availability of common allergens (cross-reactive Bet v1) (Biedermann et al. 2019; Niederberger et al. 1998; Weber 2003). These species are monoecious with typical long catkins containing only male flowers (Filbrandt-Czaja and Adamska 2018). Hazel and alder flower early in January to March, whereas birch flowers later in March to June (only in northern latitudes).

The sampling took place on the specimen in the 3 km surroundings of the campus of the Technical University of Munich at Freising/Weihenstephan (48.400292 N, 11.716874 E). Care was taken to ensure that the selected trees had a minimum distance of 15 m from buildings and varied in age, diameter, and height. From this selection, 3 hazel, 2 alder, and 2 birch tree specimen were chosen. Heights were measured with a relascope, diameters were measured with a tape measure, and ages were estimated. The selected hazels were approximately of the same age (20 years old), height (8 m), crown length (6 m), and diameter (20 cm) and had similar densities to male catkins. The two alder trees were likely of the same age (25 years old), height (15 m), crown length (13 m), and diameter (30 cm) and had about the same number of male catkins. The two birch specimens were of different estimated age (30 and 50 years old), height (15 and 25 m), crown length (13 and 23 m), and diameter (30 and 40 cm).

### Harvest and treatments of twigs

The investigation period was from the 5th of November 2018 to the 18th of April 2019. Twigs of the respective species and specimens were harvested in weekly intervals before the actual flowering (Table 1). It was assured that the harvested twigs (length 40 to 50 cm) had the required minimum number of five male inflorescences. The sampling height was 2–3 m above the ground level. After cutting, the twigs were disinfected with 90% ethanol and their basis was recut underwater to avoid disruption of water uptake. The twigs were then stored in glass bottles (capacity 100 ml) which were filled with tap water.

**Table 1:** Overview on harvested twigs including the following: species, specimen number, flowering location (indoors or outdoors); cutting date; number of twigs analyzed in the laboratory (number of twigs exclusively analyzed for allergen content in square brackets []): indoors: climate chamber (CC) and window sill (WS) subdivided into treatments control (C), plant fertilizer (PF), tissue fertilizer (TF), and outdoors: into the treatments cut and uncut; Flowering date refers to the observation date where BBCH 60 was recorded

Species	Specimen	In-/outdoors	Cutting date	Number of twigs analyzed in the laboratory	Flowering [BBCH 60]
Hazel	1 and 2	Indoors	12-06-2018	CC (5 [2] C, 4 [1] PF, 8 [2] TF), WS (5 [2] C, 3 [1] PF, 4 [2] TF)	12-13-2018
Hazel	1 and 2	Indoors	12-12-2018	CC (4 [2] C, 3 [1] PF, 7 [1] TF), WS (5 C, 4 [3] PF, 2 TF)	12-18-2018
Hazel	3	Outdoors	02-13-2019	3 [1] cut, 5 [1] uncut	02-19-2019
Alder	1 and 2	Indoors	01-21-2019	CC (1 [1] C, 4 [1] TF), WS (3 [2] C, 2 [1] TF)	01-31-2019
Alder	1 and 2	Outdoors	02-13-2019	2 [1] cut, 3 [2] uncut	03-06-2019
Alder	1 and 2	Outdoors	02-25-2019	3 [2] cut, 2 [1] uncut	03-06-2019
Alder	1 and 2	Outdoors	02-28-2019	1 [1] uncut	03-06-2019
Birch	1 and 2	Indoors	03-12-2019	CC (6 [2] C, 4 [2] TF), WS (4 C, 3 [1] TF)	03-21-2019
Birch	2	Indoors	03-29-2019	CC (3 [1] C, 3 [2] TF), WS (3 C, 2 TF)	04-04-2019
Birch	2	Indoors	04-04-2019	CC (5 [1] C), WS (5 [1] C)	04-04-2019
Birch	2	Outdoors	03-22-2019	1 [1] uncut	04-04-2019
Birch	2	Outdoors	03-23-2019	1 [1] uncut	04-04-2019
Birch	2	Outdoors	03-29-2019	2 cut, 3 [1] uncut	04-04-2019
Birch	2	Outdoors	04-04-2019	4 [2] cut, 7 [3] uncut	04-04-2019
Birch	2	Outdoors	04-06-2019	4 [1] cut, 5 [1] uncut	04-04-2019
Birch	2	Outdoors	04-09-2019	6 [1] uncut	04-04-2019

Previous experiments suggested that the usage of different fertilizers as an additive in irrigation may have influence on development of the inflorescences (AL-Kahtani and Ahmed 2012; Maksoud 2000). Therefore, hazel was treated respectively with pure water, tissue fertilizer (B5 medium—100 ml/liter), and plant fertilizer (nitrogen (N) 8%, phosphorus (P_2_O_5_) 8%, potassium (K_2_O) 6%—1 ml/liter) (Table 1). Since the first sampling series on hazel twigs did not show any difference in the development of inflorescences between the fertilizers, twigs of alder and birch were treated with pure water and tissue fertilizer only. Additionally, to each substrate, Chrysal Clear was added as remedy against mold growth.

**Fig. 1:**
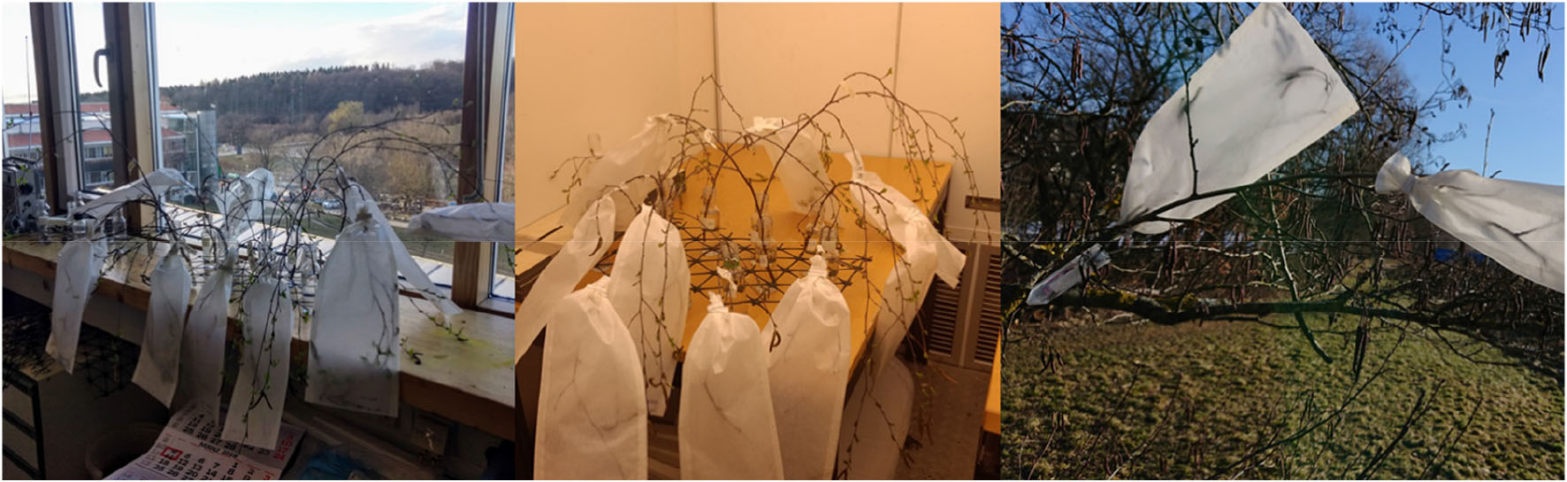
Pollen collection in glassine bags from left to right: window sill, climate chamber, outdoor conditions

The twig samples were then stored in the climate chamber which was running under a day-night cycle of 15 and 9 h, with 20 °C and 15 °C air temperature, respectively (Fig. 1). As backup in case of technical failures, a second set of samples was kept under room conditions on a window sill within the university building. Those conditions did not reflect any climate scenario; they were merely used to achieve flowering and pollen shedding.

### Comparison of cut and uncut twigs on donor trees

For hazel, alder, and birch, on-tree comparisons between cut and uncut twigs under outdoor conditions were conducted. Single twigs with a minimum of five inflorescences were cut from the donor trees at different time intervals before natural flowering (Table 1) and directly put into plastic water containers (capacity 25 ml) which were afterwards attached vertically with wires to their original position. As a control, on the respective cutting dates, the same number of uncut twigs was selected on the donor trees in the surrounding of the cut twigs. Shortly before flowering, the cut and the selected uncut branches were covered with glassine bags for the in situ collection of pollen. After the pollen emission was completed, the twigs with their glassine bags were cut.

Before separating the pollen from the anthers, the glassine bags had to be dried for approximately 1 week. Since the bags were only made of a material similar to paper, the outdoor in situ pollen collection was heavily impacted by precipitation (rain and snow) as well as by condensed water. The pollen already emitted into the bags got affected by humidity when the bags were soaked through. The inherent problem was that pollen partly burst into small pieces and after drying could not be separated from the paper anymore. To overcome this issue, we tried to cover the glassine bags additionally with plastic bags filled with a desiccant. Unfortunately, after a short time, condensation water had accumulated in the plastic bags causing similar issues as before. Still, part of the samples could be used for further laboratory analysis.

### Phenological observations and climatic parameters

During the study period from the 5th of November, 2018, to the 18th of April, 2019, we recorded phenological stages once or twice a week according to the BBCH code (Meier 2001), with a particular focus on the period with sporadically first flowers open (BBCH 60). The BBCH was recorded indoors (climate chamber and window sill) and outdoors on the sampling trees. Temperature and air humidity were continuously measured in 30 min temporal resolution by Hobo Loggers (type U23 Pro v2), always at the height of the samples.

### Laboratory analysis

After completion of flowering, the bags were inverted and gently shaken, resulting in accumulation of pollen at the bottom of the bags. After removal of the branches, the catkins/anthers were manually separated from the pollen using tweezers. The remaining pure pollen was finally weighed. For each twig, the average pollen amount per catkin and the average catkin length were determined. The weighted pollen was stored at −20 °C.

The subsequent laboratory analysis comprised the measurement of mean pollen weight per grain, protein content per grain, and allergen content. 5 mg pollen was weighed in, and then 1 ml buffer was added, and 20 μl of this mixture was given onto a slide which was then inserted into a cell counter (Bio-Rad; type TC10) for automatic counting. The average pollen grain weight was derived from the initial weight (5 mg) and the count from the instrument.

**Fig. 2:**
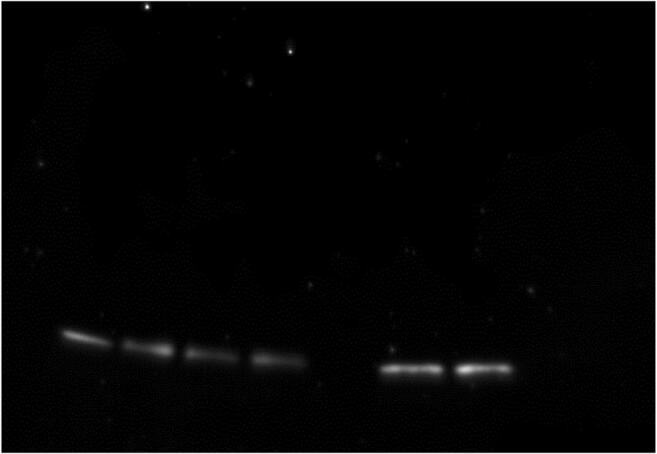
Western blot—chemiluminescence measurement for six alder samples, measurement time 100 s

Before protein and allergen quantification, the protein had to be extracted from the pollen. For this purpose, 10 mg pollen given in 1 ml buffer was shaken continuously for 3 h. The supernatant was stored at −20 °C. For protein quantification, we tested the BCA (Pierce BCA™ Protein Assay) and Bradford (Bio-Rad Protein Assay Dye Reagent Concentrate) methods in a pre-test with purification of the extract and gel electrophorese. The results of the Bradford test for protein content were in good agreement with other related studies (Ozler et al. 2009; Schäppi et al. 1997), while the results obtained with the BCA method showed much higher values. Therefore, protein quantification as protein content per pollen grain was performed using the Bradford test only. To determine allergen content, the Western blot technique was applied, which uses three different antibodies: human antibody (sera mixture of 29 patients who are allergic to Birch), monoclonal anti-human IgE antibody produced in mouse (Sigma-Aldrich), and rat anti-mouse IgG2b (–HRP conjugated, produced by The Monoclonal Antibody Core Facility at the Helmholtz Center ). After the application of all three antibodies, the allergens were detected in the last step under chemiluminescence (ECL™ select Western blotting detection reagent) (Fig. 2). Among the allergens visualized, the allergen Bet v1 showed the clearest signal as it is highly abundant. Bet v1 is cross-reactive in hazel, alder, and birch and can therefore be detected in all three species. For the calculation of Bet v1 allergen content, the signal intensity of chemiluminescence was extracted using the Fiji package from ImageJ (Rueden et al. 2017; Schindelin et al. 2012) and the protein content was determined via Ponceau S staining during Western blotting. The allergen content was then calculated by dividing signal intensity and protein content, thus it is a relative value without unit. All laboratory analyses were conducted in cooperation with the Helmholtz Center.

### Statistics

The statistical analysis was conducted in RStudio (version 3.5.1/2018-07-02). The per treatment distributions of the parameters average catkin length (cm), average pollen weight per catkin (mg), pollen weight per grain (ng), protein content per grain (ng), and allergen content (unitless) were tested by the Shapiro-Wilk test for normality. Since the parameters were not normally distributed (p-value of Shapiro-Wilk test < 0.05), non-parametric tests were further used: Student’s t-test for comparisons between two groups, the univariate ANOVA (Kruskal-Wallis test) for comparisons with more than two groups, and subsequently using pair-wise Wilcoxon tests for individual group comparison. p-values less than 0.05 were considered statistically significant. Due to the limited number of samples per treatment which could be analyzed for the pollen weight per grain (ng) and allergen content, no statistical test could be performed and thus, only differences are shown in the results.

## Results

**Fig. 3:**
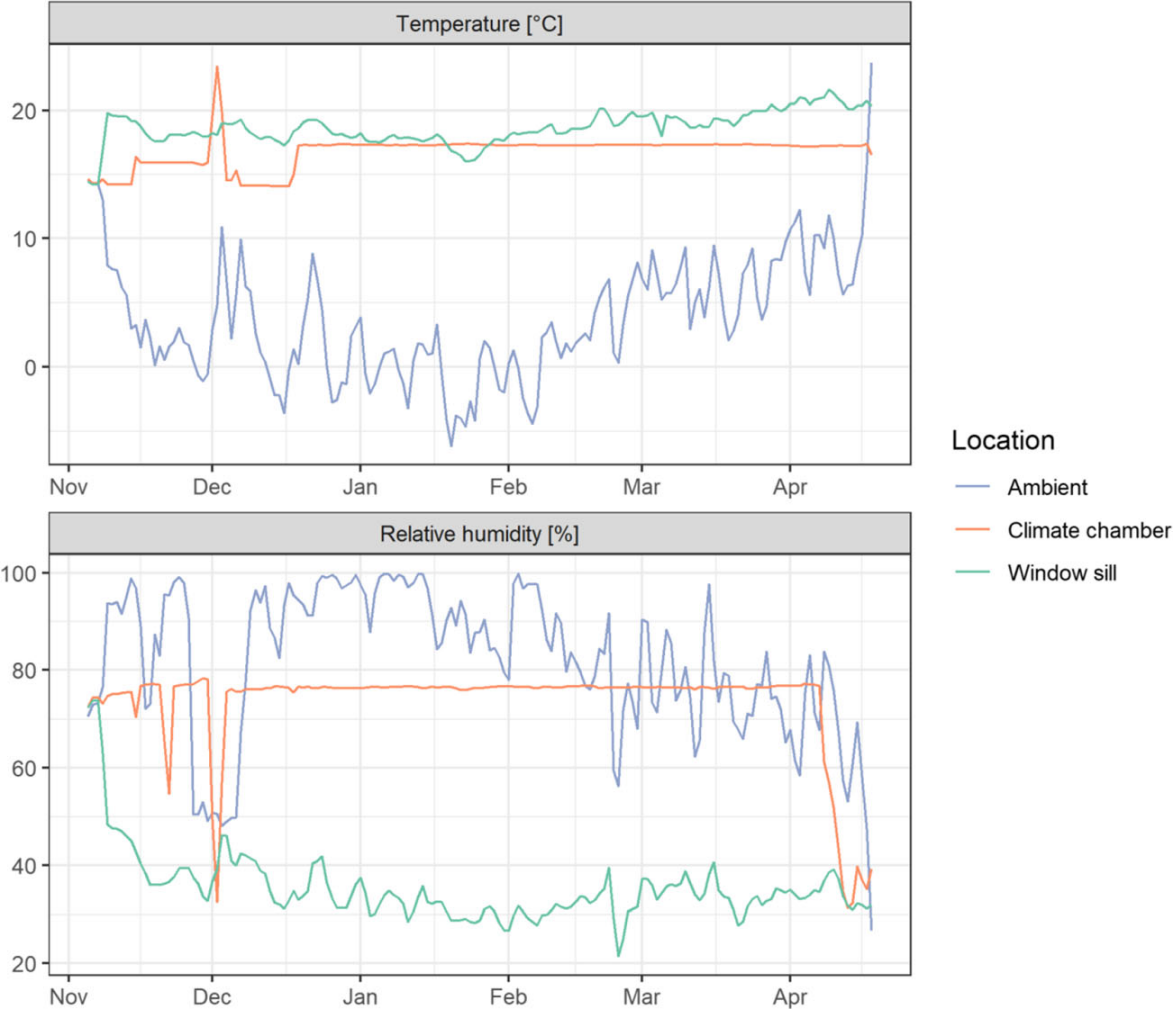
Temperature (°C) and relative humidity (%) indoors at the window sill, in the climate chamber, and outdoors during the investigation period 5th of November 2018 to 18th of April 2019

During the investigation period, temperature and relative air humidity outdoors were on average 3.7 °C and 81.9% respectively, whereby the minimum daily air temperature of −6.2 °C was reached on the 20th of January 2019 and the maximum air humidity of 99.9% on the 11th of January 2019, and the maximum of 23.6 °C and the minimum relative air humidity of 26.7% both on the 18th of April 2019. Indoors, in the climate chamber, 16.8 °C and 73.3% and on the window sill 18.6 °C and 35.3% (air temperature and humidity respectively) were recorded on average (Fig. 3).

Hazel was harvested for the indoor experiments twice on the 6th and 12th of December 2018, for the outdoor cut/uncut investigation twigs were cut/selected on the 13th of February 2019. First flowering (BBCH 60) both in the climate chamber/on the window sill was observed for the twigs cut on the 6th of December 2018 on the 13th of December 2018 and for the twigs cut on the 12th of December on the 18th of December, under outdoor conditions for both the cut and selected twigs on the 19th of February 2019 (Table 1).

**Fig. 4:**
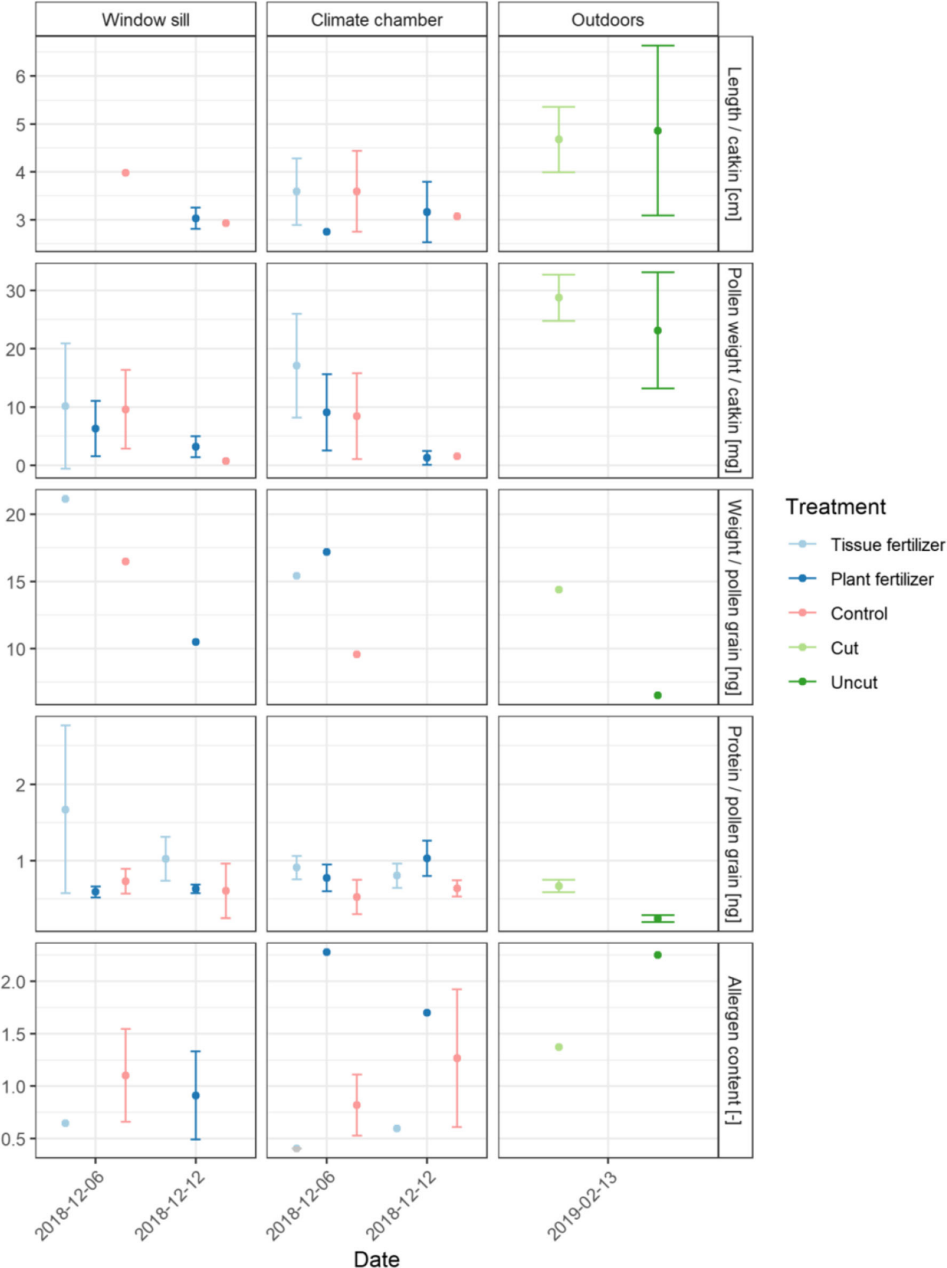
Results of the laboratory analysis for hazel: length per catkin (cm), average pollen grain weight (ng), protein content per pollen grain (ng), and allergen content (unitless) for the respective indoor flowering locations window sill and climate chamber, as well as outdoors under the treatments tissue fertilizer, plant fertilizer, control, cut and uncut for different harvesting dates; bars represent standard deviation according to the sample size

Between the two fertilizers and the control, significant differences were found in protein content (p<0.057), and no significant differences in length per catkin (p=0.28) and in pollen weight per catkin (p=0.11) (Fig. 4). There were no significant differences in any of these parameters between the two harvesting dates (the 6th and 12th of December 2018). In comparison to the outdoor conditions, the catkin length and the weight per pollen grain were significantly smaller in the climate chamber/window sill (p=0.031 and p=0.0013). Under outdoor conditions, the cut treatment showed a higher average pollen grain weight (14.4 ng) than the uncut treatment (6.5 ng). For the allergen content, an opposite behavior was observed. The uncut treatment had higher allergen content (2.3) than the cut treatment (1.7).

**Fig. 5:**
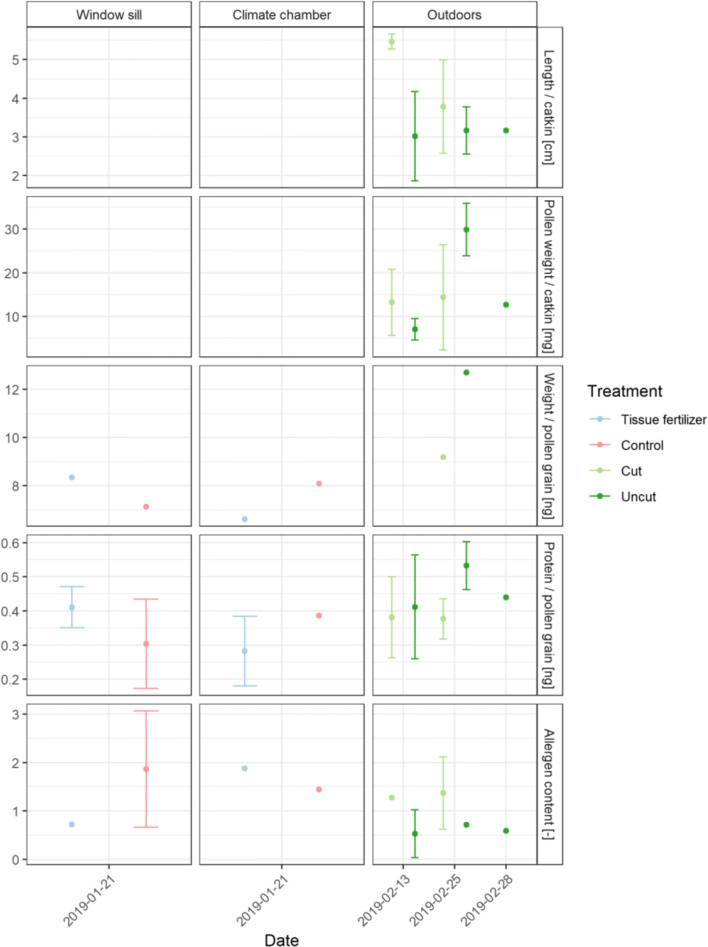
Results of the laboratory analysis for alder: length per catkin (cm), average pollen grain weight (ng), protein content per pollen grain (ng), and allergen content (unitless) for the respective flowering locations window sill and climate chamber, and outdoors under the treatments tissue fertilizer, plant fertilizer, control, cut and uncut for different harvesting dates; bars represent standard deviation according to the sample size, missing data for length per catkin (cm) and protein content per pollen grain (ng) for the cutting date 21th of January 2019

Alder was harvested on the 21st of January 2019 for indoor investigation; outdoor twigs were cut/selected on the 13th, 25th, and 28th of February 2019. Flowering in the climate chamber/window sill was recorded on the 31th of January 2019, under outdoor conditions on the 6th of March 2019. Between the treatments tissue fertilizer and the control, no significant differences were found (Fig. 5). There were significant differences in the protein content between climate chamber/window sill and outdoor conditions (p=0.046). For the catkin length and the pollen weight in the climate chamber/window sill, no results were available. Under outdoor conditions, the average pollen grain weight (ng) was higher for the uncut (12.7 ng) than the cut treatment (9.2 ng).

**Fig. 6:**
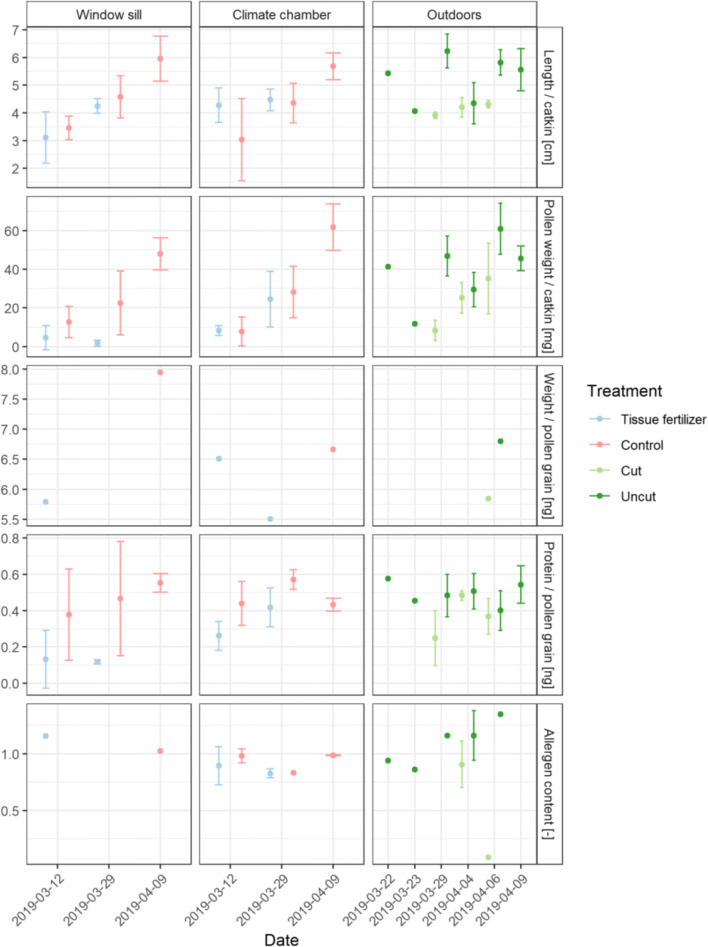
Results of the laboratory analysis for birch: length per catkin (cm), average pollen grain weight (ng), protein content per pollen grain (ng), and allergen content (unitless) for the respective flowering locations window sill and climate chamber, and outdoor conditions under the treatments tissue fertilizer, plant fertilizer, control, cut and uncut for different harvesting dates; bars represent standard deviation according to the sample size

Birch was harvested on the 12th and 29th of March and the 9th of April 2019; outdoor twigs were cut/selected on the 22nd, 23rd, and 29th of March and on the 4th, 6th, and 9th of April 2019. Flowering in the climate chamber/window sill from the twigs cut on the 12th of March was observed on the 21th of March 2019 and for the two later cuttings on the 29th of March and 4th of April flowering could be monitored on the 4th of April. Outdoors flowering was observed on the 4th of April 2019. There were no significant differences between the treatments tissue fertilizer and control in catkin length, pollen weight per catkin, and protein content per grain (Fig. 6). Under controlled conditions in the climate chamber/window sill, later, harvesting revealed higher values for the catkin length, the pollen weight per catkin, and the protein content. The results were significant for the parameters catkin length (p=0.0054), pollen weight per catkin (p=0.023) but not for the protein content (p=0.27). For the comparison between outdoor and the climate chamber/window sill, significant differences were found for the catkin length (p=0.031) and pollen weight per catkin (p=0.0064) but not for the protein content (p=0.09). The average pollen grain weight was in the uncut treatment higher (6.8 ng) than in the cut treatment (5.9 ng).

## Discussion

The main objective of this study was to establish the twig method in climate chambers for the investigation of flowering phenology and pollen characteristics of allergenic shrub/tree species. In order to evaluate whether the cutting of twigs influenced flowering, pollen emission, and pollen characteristics, a comparison of cut and uncut twigs outdoors on donor trees was conducted. The studied tree species hazel, alder, and birch showed in most cases (except birch on the 29th of March 2019) only marginal differences in the studied parameters (except average pollen grain weight and allergen content) for the outdoor comparison between cut and uncut branches. For allergen content and pollen weight per pollen grain, a higher sample number would be required for a solid assessment. Flowering occurred on the same day, the pollen amount emitted per catkin was similar and differences in the pollen characteristics were minor and not significant. Variations in the results for different twig selecting dates (uncut treatment having the same harvesting date) can be explained by a varying location of the selected twigs on the respective donor tree. Based on the results of the cut and uncut comparison on the donor trees, the usage of cut twigs can be recommended as appropriate for flowering and pollen investigations.

Furthermore, it was studied whether the usage of different fertilizers as an additive in irrigation has an influence on the vitality of the inflorescences and other observed parameters. Dormant twigs cut before natural flowering from hazel, alder, and birch were kept indoors under constant conditions in climate chamber or room condition (window sill) until full flowering and pollen emission. The respectively added plant and tissue fertilizer in contrast to other studies (AL-Kahtani and Ahmed 2012; Maksoud 2000) neither affected plant vitality nor the observed parameters. The weekly exchange of water seemed to be sufficient for proper inflorescence development as potentially occurring mold (Criado et al. 2005) could in the meantime be prevented by the addition of a remedy against fungal growth.

In order to study the possibility of using climate chamber experiments as proxy for outdoor manipulations, twigs were harvested in different intervals (see Table 1) before natural outdoor flowering and were kept in the climate chamber/room conditions for flowering. In comparison to the outdoor conditions (average temperature 3.7 °C), a much earlier flowering was observed in the climate chamber (average temperature 16.8 °C) for hazel (−69 days), alder (−35 days), and birch (−15 days), ordered by their harvesting dates beginning of December, end of January, and beginning of March. Wang et al. (2020) observed similar trends in a climate chamber experiment on branches of six Asian woody species, where a 3 °C increase in spring temperature resulted in flowering advanced by 2.3 to 36.1 days, depending on the species. In the present study, an impact of the flowering time could be observed on the pollen amount produced, and on pollen characteristics for all investigated species. In the climate chamber, later branch harvesting and thus later flowering resulted in higher pollen production per catkin, catkin length, and protein content. This could be seen in the respective results from the climate chamber/window sill as compared to the outdoor conditions. For birch, the same dependency could be seen for the different harvesting dates between the 12th of March and the 9th of April. A study on birch from (Buters et al. 2010) showed a similar dependency for the allergen content, it strongly increased in the days before flowering. Related studies on birch have shown that climate warming can affect leaf size and the number of shoots developed (Hofgaard et al. 2010), as well as leaf phytochemistry (Jamieson et al. 2015).

Since the pollen weight per grain was not investigated for each harvesting date separately (due to lack of pollen amount), further experiments would be needed. The generally considerably lower relative humidity on the window sill (room temperature) in comparison to the climate chamber seemed not to affect the flowering timing nor pollen characteristics. It cannot be ruled out that there are still drivers that have not been accounted for but still influence flowering in the climate chamber (Wolkovich et al. 2012).

The taken twig samples from different harvesting dates were able to flower in the climate chamber/window sill. Flowering in the climate chamber/window sill occurred 5–10 days after harvesting of the twigs. The respective five catkins per twig sample produced a sufficient amount of pollen (>10 mg) for further analysis in the laboratory. These results confirm that the twig methods in climate chambers can be a proxy for outdoor manipulations and can therefore be used for various experimental setups in pollen research (different climatic scenarios for temperature, air humidity, ozone, CO_2_) (Primack et al. 2015; Vitasse and Basler 2014).

A recent study by Ettinger et al. (2020) pointed out the dominant influence of winter temperatures on spring phenology and how manipulation experiments can contribute to its further exploration. Branch experiments can simulate variation in chilling length using different harvest dates prior to actual flowering or higher temperatures in the climate chamber/window sill can be used to study the effect of accelerated warming (forcing) (see e.g. Menzel et al. 2020a, b).

## Conclusion

The twig method in climate chambers has been already well established for observations on phenology, budburst, and leaf unfolding. The presented study could illustrate that the twig method in climate chambers is also well applicable for investigations on flowering and pollen characteristics. In situ comparisons on outdoor shrubs/trees revealed no significant difference between male catkins from cut and uncut twigs in their time of flowering and pollen characteristics. The addition of different fertilizers during the irrigation of the cut twigs did not show a significant effect on the vitality of the inflorescences nor the pollen characteristics. Twigs from hazel, birch, and alder were cut in different intervals before natural flowering and then kept under controlled conditions in the climate chamber/window sill for flowering. It could be seen that accelerating warming influences flowering and the pollen characteristics catkin length, pollen emission per catkin, and protein content. The twig samples from all cutting dates were able to produce an appropriate amount of pollen for the subsequent laboratory analysis. This shows that the twig method in climate chambers can be used as a proxy for outdoor manipulations in pollen research.
